# Heart failure in nursing home residents; a cross-sectional study to determine the prevalence and clinical characteristics

**DOI:** 10.1186/s12877-015-0166-1

**Published:** 2015-12-16

**Authors:** Mariëlle A. M. J. Daamen, Jan P. H. Hamers, Anton P. M. Gorgels, Hans-Peter Brunner-La Rocca, Frans E. S. Tan, Marja P. van Dieijen-Visser, Jos M. G. A. Schols

**Affiliations:** Department of Health Services Research, Research School Caphri, Maastricht University, Maastricht, The Netherlands; Department of Cardiology, Maastricht University Medical Centre, Maastricht, The Netherlands; Department of Methodology and Statistics, Research School Caphri, Maastricht University, Maastricht, The Netherlands; Department of Clinical Chemistry, Maastricht University Medical Centre, Maastricht, The Netherlands; Department of Family Medicine, Research School Caphri, Maastricht University, Maastricht, The Netherlands

**Keywords:** Heart failure, Nursing home residents, Diagnosis, Prevalence

## Abstract

**Background:**

Heart failure (HF) is expected to be highly prevalent in nursing home residents, but precise figures are scarce. The aim of this study was to determine the prevalence of HF in nursing home residents and to get insight in the clinical characteristics of residents with HF.

**Methods:**

The study followed a multi-centre cross-sectional design.

Nursing home residents (*n* = 501) in the southern part of the Netherlands aged over 65 years and receiving long-term somatic or psychogeriatric care were included in the study. The diagnosis of HF and related characteristics were based on data collected from actual clinical examinations (including history, physical examination, ECG, cardiac markers and echocardiography), patient records and questionnaires. A panel of two cardiologists and a geriatrician ultimately judged the data to diagnose HF.

**Results:**

The overall prevalence of HF in nursing home residents was 33 %, of which 52 % had HF with preserved ejection fraction. The symptoms dyspnoea and oedema and a cardiac history were more common in residents with HF. Diabetes mellitus, chronic obstructive pulmonary disease (COPD) were also more prevalent in those with HF. Residents with HF had a higher score on the Mini Mental State Examination. 54 % of those with HF where not known before, and in 31 % with a history of HF, this diagnosis was not confirmed by the expert panel.

**Conclusion:**

This study shows that HF is highly prevalent in nursing home residents with many unknown or falsely diagnosed with HF. Equal number of HF patients had reduced and preserved left-ventricular ejection fraction.

**Trial registration:**

The Netherlands National Trial Register NTR2663 (27-12-2010)

## Background

In Western countries, heart failure (HF) is common in older people. The prevalence of HF increases up to 17.4 % at the age of 85 years and more [1]. It is a growing problem as the population ages and survival rates after cardiovascular events increase [2]. In addition, there is a longer exposure to risk factors for HF and age-related changes [3]. Various factors such as older age, hypertension, diabetes mellitus (DM), obesity and coronary artery disease (CAD) have been described as risk factors for developing HF [4, 5].

Early diagnosis and treatment of HF may prevent progression and lead to improvement of symptoms and quality of life [6, 7], which are especially important in the care of nursing home residents. These residents can be described as old and considerably disabled persons, with either chronic somatic diseases or progressive dementia beyond the range of home care services [8]. The diagnosis of HF in nursing home residents is however challenging, due to atypical signs and symptoms, cognitive impairment, immobility, polypharmacy and misinterpretation of symptoms due to co-morbidities [9, 10]. Importantly, these residents often do not undergo proper diagnostics as recommended by the guidelines [11]. As a consequence, the prevalence of HF in such residents may be significantly underestimated, but also overestimated due to misinterpretation of symptoms corresponding to comorbidities. A systematic review by Daamen et al. showed that the prevalence of HF in nursing home residents is estimated to be 15–25 % [12]. The diagnosis of HF was, however, based on information derived from medical records only in all but one study included. In this study by Butler et al., the diagnosis of HF was made after a clinical examination, resulting in an HF prevalence as high as 45 % [13]. However, the prevalence in UK long-term care facilities was recently reported to be much lower when also using echocardiography (i.e. 23 %) [14]. Thus, there is significant uncertainty regarding the proper diagnosis of HF in nursing home residents. The aim of this study was, therefore, to determine the prevalence and clinical characteristics of HF in Dutch nursing home residents, based on an onsite comprehensive HF assessment, including not only medical history, medication and clinical assessment, but also ECG, echocardiography and biomarkers, and final diagnosis was made by an expert panel.

## Methods

This study followed a multi-centre cross-sectional design as previously published [15]. The study protocol complied with the declaration of Helsinki and has been granted approval from the Medical Ethics Committee of Maastricht University/Academic Hospital Maastricht (NL33281.068.10/MEC10-3-074). The study has been registered in the Netherlands National Trial Register (NTR2663).

### Setting and participants

In the southern parts of the Netherlands, long-term care is covered by five organizations providing nursing home care, with an overall number of approximately 4.500 nursing home residents. Nursing home care in the Netherlands is provided by teams of registered nurses, nurse assistants, paramedical professionals and nursing home physicians, who are employed by the nursing homes themselves [16]. The types of care offered by nursing homes include long-term care (somatic or psychogeriatric), rehabilitation, respite care, palliative (or hospice) care, consultation and advice and crisis intervention [8].

Nursing home residents at 28 locations allocated to these five long-term care organizations were asked to participate in the study (*n* = 1 920). To complete the study within the specified time period (January 2011 to June 2013) and for practical and logistical reasons, nursing home residents living in the facilities with the highest number of residents were first recruited within each care home organization. The number of locations involved per organization differed from 3 to 8. Residents were eligible if they received chronic somatic care or psychogeriatric care and were over 65 years of age. Residents who received palliative care or were admitted for short-term rehabilitation (staying less than 2 months) were excluded. Informed written consent was obtained from the residents themselves or from their legal representatives in the case of psychogeriatric disorder, or residents with aphasia.

Based on our systematic review, the expected prevalence of HF was 20 % with a range of 15–45 % [12]. For the calculation of the required sample size, it seemed reasonable to assume that the estimated prevalence of HF in nursing home residents would be within the range *p* = 0.20 to *p* = 0.40 with a confidence interval width of 25 % of the estimated prevalence. Accordingly, 368 (if *p* = 0.40) to 983 residents (if *p* = 0.20) [15] should be included in the study for sufficient power as described [15].

### Measurements and materials

#### Demographic data and clinical characteristics

Data regarding general and clinical characteristics were gathered for all participating residents. These included age, gender and symptoms and signs of HF (dyspnoea, orthopnoea, palpitations, paroxysmal nocturnal dyspnoea (PND), fatigue, increased weight, pulse rate, blood pressure, increased jugular venous pressure, hepatojugular reflux, right ventricular pulsations, hepatic pulsations, hepatomegaly, oedema, palpable apex, displacement of the apex, third heart sound, murmur, pulmonary rales, pleural effusion). Moreover, information regarding cardiac history (hypertension, myocardial infarction, arrhythmia, CAD, valvular heart disease, coronary bypass graft, pacemaker, pre-existing heart failure), co-morbidity, cardiovascular risk factors (Body Mass Index (BMI), hypercholesterolemia, DM and smoking) and medication (cardiac and non-cardiac medication) were collected. Patients underwent a Mini Mental State Examination (MMSE), blood sampling, a standard 12-lead electrocardiogram (ECG) and an echocardiogram. Doppler-echocardiography was performed according to current standards from parasternal, apical and epigastric views [17].

#### Study procedure of the heart failure assessment

First, a nursing home physician (NHP) assessed medical history and performed physical examination. Before the start of the study, this NHP (3–4 per organization) had received a refresher course on diagnosing HF and performing a structured physical examination. The training included a review of signs and symptoms of HF followed by bedside teaching (3 h), a lecture on ECG findings in relation to HF (2 h) and a visit to the outpatient HF clinic of the Maastricht University Medical Centre (3 h). Two research nurses and an NHP/researcher were responsible for recording the ECGs, collecting the blood samples, gathering data from the medical records and filling in the questionnaires. Qualified (fellow) cardiologists recorded an echocardiogram on site. The entire assessment was carried out on site in the participating nursing homes with a mean time span of four weeks to complete data collection per resident.

The actual history and physical examination were recorded on a client record file developed for this study. Somatic residents answered the history-related questions and for psychogeriatric residents, these questions were answered by the nurses responsible for their daily care or the main family caregiver of the resident. N-terminal of the prohormone brain natriuretic peptide (NT-pro BNP), haemoglobin and serum creatinine concentrations were analysed at the clinical chemistry laboratory of the Maastricht University Medical Centre on an Elecsys 2010 (Roche Diagnostics, Mannheim, Germany). A standard 12-lead ECG was made using the Cardiovit AT-10 plus (Schiller, Switzerland). The following (possibly HF-related) ECG findings were recorded: sinus tachycardia (heart rate > 100 beats/min), sinus bradycardia (heart rate < 60 beats/min), pathological Q-waves, atrial fibrillation, ventricular arrhythmia, different degrees of AV-block, low voltage of the QRS complexes (<5 mm in the frontal plan), QRS widening (>0.12 ms) due to right bundle branch block, left bundle branch block or non-specific widening and left (LV) and/or right ventricular (RV) hypertrophy [18]. The echocardiogram was performed using a Philips CX50 Compact Xtreme Ultrasound System (Philips, the Netherlands). The following parameters were measured using standard views (Lang); LV end-diastolic and end-systolic diameter, LV wall thickness, LV function indicators such as fractional shortening and LV ejection fraction, diastolic function variables such as E/A and E/E’ ratio, left atrial size, valvular structure and function, tricuspid regurgitation velocity peak as an indicator of RV systolic pressure, RV dilatation and/or hypertrophy and dysfunction and vena cava inferior width and respiratory variation as a marker of venous overload. A LV ejection fraction of ≥ 50 % was regarded as preserved ejection fraction [19].

#### Diagnosis of heart failure

An expert team of two cardiologists and a geriatrician were responsible for the final diagnosis of HF using all data collected by the NHP, research nurse and (fellow) cardiologist, based on the current ESC guidelines [20].

1. Signs and symptoms of HF
2. Presence of structural or functional cardiac abnormalities.

The members of the expert team judged all files independently. First the data were judged on the presence of signs and symptoms. Subsequently, the measurements of the echocardiography and the ECG were used to assess functional and/or structural cardiac abnormalities, compatible with the diagnosis of HF. In the absence of current signs and symptoms, medical history and medication provided important information to make the diagnosis of HF. In residents without an echocardiogram or in the case of uncertainty regarding the diagnosis, the NT-pro BNP value, ECG findings, medical history and medication (in that order) were considered for the final diagnosis.

When there was mutual agreement on the diagnosis, the file was closed. All files with disagreement were discussed in the presence of all three members of the expert team to reach a consensus on the final diagnosis.

### Statistical analysis

Statistical analyses were performed using IBM SPSS statistics software version 20 and included descriptive frequency distributions for all variables. Differences between groups were tested using Student’s *t*-tests or analysis of variance (ANOVA) for continuous variables and Chi-square test (cross-table analysis) and multivariable logistic regression analysis for discrete variables, respectively, with a level of significance of p < 0.05 for all variables. The selection of variables included in the multivariable logistic regression analysis was based on the literature and expert opinion. These variables were gender, CAD, BMI, DM, chronic obstructive pulmonary disease (COPD), haemoglobin, creatinine, heart rate, systolic blood pressure, pre-existing HF, arrhythmia, valvular heart disease, [21, 22]. To avoid circular reasoning the variables dyspnoea, signals of right sided HF and NT-pro BNP were not added to the model, because these were used to diagnose HF.

## Results

### Study sample

**Fig. 1:**
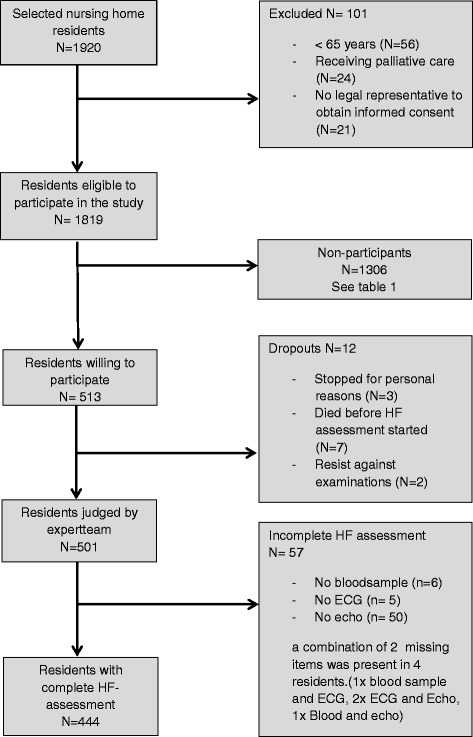
Flow chart of residents participation

Of the residents fulfilling the inclusion criteria, 27 % of those or their legal representatives agreed to participate. Main reasons for not participating included that residents considered themselves too old or believed that the investigations were too burdensome. The expert team eventually assessed 501 client record files and decided on the diagnosis of HF. HF assessment (medical history, physical examination, blood sampling, ECG and echo) was completed for 87 % of the participating residents (see Fig. 1 for further details). Missing blood samples occurred because of the inability to puncture the vein. Some ECG’s could not be made due to resistance (*n* = 3) and extreme tremor in the limbs (*n* = 2). Missing outcomes of echocardiography were mainly caused by poor image quality due to e.g. obesity and in case of COPD. In a minority of cases (*n* = 10) psychogeriatric residents resisted against the echocardiography.

The demographic characteristics, age, gender and ward type showed no clinical significance differences for both participants and non-participants (see Table 1).

**Table 1:** Basis characteristics of participants and non-participants

Characteristics		Participants (*n* = 513)	Non-participants (*n* = 1306)*
Age (years)	65–74	69 (14 %)	116 (11 %)
	75–84	238 (46 %)	436 (43 %)
	85+	206 (40 %)	464 (46 %)
	Mean (SD, range)	82 (7, 65–100)	83 (7, 65–108)
Gender	Male	184 (36 %)	376 (29 %)
	Female	329 (64 %)	930 (71 %)
Ward	Psychogeriatric care	336 (65 %)	889 (68 %)
	Somatic care	177 (35 %)	417 (32 %)

*Age available for *n* = 1016 non-participants

### The prevalence of heart failure

**Fig. 2:**
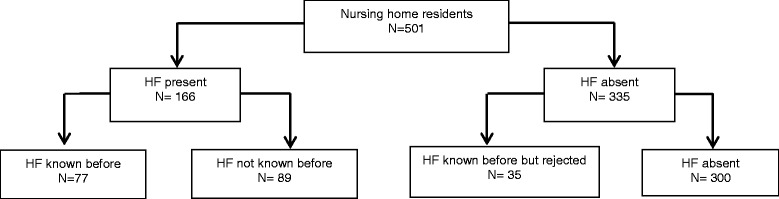
Prevalence of HF compared with HF previously recorded in medical files

The prevalence of HF in nursing home residents was 33 % with a 95 % confidence interval of 29 to 37 %, of which 54 % were not previously known (Fig. 2). HF was more often diagnosed in the somatic group than in psychogeriatric residents. The prevalence was practically similar in men and women. Reduced LV ejection fraction was present in 64 and preserved ejection fraction in 70 cases. Asymptomatic systolic dysfunction of the left ventricle (LVEF < 50 without symptoms, stage B) was found in 27 residents (see also Table 2) [20].

**Table 2:** Presence of heart failure in nursing home residents

Variabele		HF absent	HF present	(95 %-CI) of perc. HF	HFrEF^a^	HFpEF^a^	Asymptomatic HF with rEF^b^
Total		335 (67 %)	166 (33 %)	(29 %, 37 %)	64 (48 %)	70 (52 %)	27 (10 %)
Gender	Male	118 (66 %)	61 (34 %)	(30 %,38 %)	29 (58 %)	21 (42 %)	12 (44 %)
	Female	217 (67 %)	105 (33 %)	(29 %,37 %)	35 (42 %)	49 (58 %)	15 (56 %)
Ward	Psychogeriatric care	227 (70 %)	99 (30 %)	(26 %,34 %)	36 (45 %)	44 (55 %)	19 (70 %)
	Somatic care	108 (62 %)	67 (38 %)	(34 %,42 %)	28 (52 %)	26 (48 %)	8 (30 %)

HFrEF heart failure with reduced ejection fraction, HFpEF heart failure with preserved ejection fraction, rEF reduced ejection fraction

^a^Echo data about left ventricular ejection fraction (LVEF) available for 134 of the 166 residents with HF

^b^Echo data about LVEF available for 271 of the 335 residents without HF

HF was recorded in the medical history of 112 residents. The diagnosis of HF was rejected in 31 % of those residents. Figure 2 shows an overview of the prevalence of HF compared with HF previously recorded in medical files.

### Clinical characteristics

As depicted in Table 3, residents with HF were older, more likely to have complaints of orthopnoea and palpitations, more often had a cardiac history of myocardial ischemia, myocardial infarction and arrhythmia and more often displayed signs of an increased jugular venous pressure, pulsations of the right ventricle, oedema, murmurs and pulmonary rales. A higher number of nursing home residents with HF had diabetes mellitus, COPD and/or less cognitive disorders. However, none of these factors were sufficiently sensitive or specific for diagnosing or excluding heart failure. Regarding the cardiac risk factors (hypercholesterolemia, smoking and BMI), there were no differences between the study groups with and without HF.

**Table 3:** Demographic and clinical characteristics

Variables	Total	HF-	HF+	*p*-value
	*N* = 501	*N* = 335	*N* = 166	
Age (years) mean (SD)	82 (7)	81 (7)	84 (6)	<.001
Gender, male n (%)	179 (36)	118 (35)	61 (37)	0.738
psychogeriatric n (%)	326 (65)	227 (68)	99 (60)	0.073
Symptoms				
NYHA class n (%) *n* = 494				
1	301 (60)	235 (70)	66 (40)	<.001
2	108 (22)	55 (16)	53 (33)	<.001
3	57 (11)	26 (8)	31 (19)	0.001
4	28 (6)	15 (5)	13 (8)	0.203
Orthopnoea n (%) *n* = 486	62 (12)	29 (9)	33 (20)	0.001
Palpitations n (%) *n* = 484	70 (14)	27 (8)	43 (26)	<.001
PND n (%) *n* = 486	40 (8)	22 (7)	18 (11)	0.033
Fatigue n (%) *n* = 492	183 (37)	113 (34)	70 (42)	0.178
Weight gain n (%) *n* = 480	97 (19)	69 (21)	28 (17)	0.522
Physical examination				
Anemic n (%) *n* = 500	28 (6)	17 (5)	11 (7)	0.609
Cyanotic n (%) *n* = 500	1 (0,2)	1 (0,3)	1 (0,6)	0.284
Dyspnoea n (%) *n* = 497	82 (16)	43 (13)	39 (24)	0.004
Pulse rate mean (SD) *n* = 500	73 (14)	72 (12)	74 (17)	0.069
Systolic blood pressure mean (SD) *n* = 498	139 (26)	141 (26)	136 (25)	0.030
Diastolic blood pressure mean (SD) *n* = 498	75 (14)	75 (13)	74 15)	0.219
Increased VJ pressure n (%) *n* = 481	128 (26)	66 (20)	166 (37)	<.001
HPR n (%) *n* = 468	26 (5)	16 (5)	10 (6)	0.570
Pulsations RV n (%)	61 (12)	30 (9)	31 (19)	0.002
Pulsations liver n (%)	18 (4)	11 (3)	7 (4)	0.597
Hepatomegaly n (%) *n* = 484	35 (7)	20 (6)	15 (9)	0.298
Oedema n (%) *n* = 500	275 (55)	166 (50)	109 (66)	<.001
Ictus palpable n (%) *n* = 494	158 (32)	102 (30)	56 (34)	0.628
Third heart sound n (%) *n* = 467	17 (3)	10 (3)	7 (4)	0.746
Murmur n (%) *n* = 473	185 (37)	110 (33)	75 (45)	0.019
Irregular heartbeats n (%) *n* = 498	153 (31)	66 (20)	87 (52)	<.001
Rales n (%) *n* = 459	151 (30)	75 (22)	76 (46)	<.001
Pleural effusion n (%) *n* = 412	34 (7)	15 (5)	19 (11)	0.008
Cardiac history				
Hypertension n (%)	236 (47)	146 (44)	90 (54)	0.025
Myocardial infarction n (%) *n* = 490	81 (16)	37 (11)	44 (27)	<.001
Arrhythmia n (%) *n* = 476	132 (28)	54 (17)	78 (50)	<.001
Coronary ischemia n (%) *n* = 490	113 (23)	57 (17)	56 (34)	<.001
Valvular heart disease n (%) *n* = 495	38 (8)	13 (4)	25 (15)	<.001
Coronary bypass graft n (%) *n* = 496	40 (8)	17 (5)	23 (14)	0.003
Pace maker n (%) *n* = 493	19 (4)	8 (2,4)	11 (7)	0.059
Heart failure in history n (%)	112 (22)	35 (10)	77 (46)	<.001
Blood sample				
NT- pro BNP mean (IQR) *n* = 493 nmol/L	138 (3–4130)	61 (3–3427)	292 (13–4130)	<.001
Creatinine μmol/l mean (SD) *n* = 493	88 (51)	82 (44)	99 (61)	<.001
Haemoglobin mmol/l mean (SD) *n* = 467	8,0 (0,90)	8,1 (0,87)	7,8 (0,93)	0.002
Co-morbidity				
Diabetes mellitus n (%)	107 (21)	63 (19)	44 26)	0.048
COPD n (%)	83 (17)	45 (13)	38 (23)	0.007
CVA n (%)	207 (41)	131 (39)	76 (46)	0.153
Cardiac risk factors				
BMI mean (SD) *n* = 488	25 (5)	25 (5)	26 (5)	0,224
Hypercholesterolemia n (%) *n* = 444	130 (26)	88 (26)	42 (25)	0.209
Smoking n (%) *n* = 492	68 (14)	46 (14)	22 13)	0.358
Cognitive function				
MMSE mean (SD) *n* = 477	14 (9)	13 (9)	16 (8)	0.011

*NYHA* New York Heart Association, *PND* paroxysmal nocturnal dyspnoea, *HPR* hepatojugular reflux, *RV* right ventricle, *NT-proBNP* N-terminal of prohormone brain natriuretic peptide, *COPD* chronic obstructive pulmonary disease, *CVA* cerebrovascular accidents, *BMI* body mass index, *MMSE* mini mental state examination

The independent predictors of prevalent HF entered in the multivariable logistic regression model are presented in Table 4. The non-significant variables (*p* > 0.05) were removed stepwise using the backward procedure. The final model showed that the variables, CAD, arrhythmia, age, creatinine and HF in history being significant in univariate analysis were independent factors associated with the presence of HF.

**Table 4:** Multivariable logistic regression analysis

	Final model							
Variable	OR Ratio	95 %-CI		*p*-value	OR	95 %-CI		*p*-value
Gender	0.98	0.58	1.65	0.92				
Coronary artery disease	2.24	1.36	3.68	.002	2.10	1.29	3.40	0.003
Body Mass Index (kg/m^2)^	1.03	0.98	1.08	0.29				
Diabetes mellitus	1.09	0.62	1.91	0.77				
COPD	1.15	0.63	2.10	0.64				
Haemoglobin (mmol/l)	0.84	0.65	1.11	0.22				
Arrhythmia	3.80	2.32	6.23	<.001	4.12	2.55	6.65	<.001
Age (years)	1.08	1.04	1.12	<.001	1.07	1.04	1.11	<.001
Creatinine (μmol/l)	1.01	1.00	1.01	0.06	1.01	1.00	1.01	0.026
Heart failure in history	5.46	3.23	9.24	<.001	5.97	3.58	9.95	<.001
Valvular heart disease	0.92	0.72	1.18	0.52				
Heart rate (beats/min)	1.01	0.99	1.03	0.26				
Systolic blood pressure (mmHg)	1.00	0.99	1.01	0.43				

## Discussion

In this study, the prevalence of HF was 33 % for all participating nursing home residents, of which more than half were previously undiagnosed and a previous diagnosis of HF was rejected in almost one third. HFpEF and HFrEF were equally prevalent. Therefore, the hypothesis that HF is highly prevalent in nursing home residents is confirmed, and the prevalence was clearly higher than among older persons in the general population [1].

The prevalence of 45 % found in a study by Butler et al.[13] in which HF was diagnosed in nursing home residents after physical examination by a geriatrician, is higher than in our study. However, the limitations of that study were the lack of a clear description of HF and the lack of further diagnostics such as an echocardiogram as recommended by the guidelines [20]. Therefore, the presence of HF may be overestimated, which is in line with other studies of a similar population by Hancock et al.[14] and Barents et al. [23] where the prevalence of HF was much lower (i.e. 23 %). The HF assessment and definition of HF in these studies were similar to our study. The demographic and clinical characteristics of the residents were also comparable to our study. There is no obvious explanation as to why the prevalence rate in our study was somewhat higher. The difference between the studies of Hancock et al. [14] and Barents et al. [23] and our study is the confirmation of the diagnosis of HF by an expert team of two cardiologists and a geriatrician. In their studies, there were individual decisions by two cardiologists and no panel discussions.

Residents with HF had less cognitive disorders; a result which is not in line with studies in the literature where patients with HF suffer more often from cognitive disorders [24, 25]. This might be explained by the fact that there is a difference in study sample. Our nursing home residents are a specific group of older persons, where a high number suffers from cognitive disorders as the primary diagnosis. However analysis of the MMSE score in the subgroup of somatic residents still shows a higher MMSE score in residents with HF.

In the study by Hanock et al. [14], more patients (i.e. app. two-third) with HFpEF were observed as compared to our study. HFpEF was equally common as HFrEF in our study, and as expected, more prevalent in women [4]. In large cohorts of the general population, the frequency of HFrEF versus HFpEF was also the same with approximately half having reduced and half having preserved LVEF [26, 27], although average age was lower than in our cohort of nursing home residents (i.e. app 75 years). Asymptomatic left ventricular systolic dysfunction was additionally found in 10 % of the residents in our study. Taken together, there was a substantial number of residents with reduced LVEF, i.e. approximately one fourth of all residents investigated in this study. Compared with the study by Hancock et al.[14], this is a higher percentage of residents with reduced LVEF and could explain the difference in the prevalence of HF. HFrEF is better defined than HFpEF and particularly asymptomatic diastolic dysfunction in such an elderly population is difficult to determine. Therefore, direct comparison of patients with reduced LVEF between different cohorts may be more reliable despite some inaccuracy in measurement of LVEF by echocardiography [28]. Nevertheless, it obviously remains to be determined how many of the patients with asymptomatic reduced LVEF will develop signs and symptoms of HF over time.

Multivariable analysis showed that factors such as arrhythmia, CAD, age, a history of pre-existing HF were associated with the presence of HF. Interestingly, there was no association found with gender, which may be a risk factor for the development of HF in the general population [29]. There were no other studies in nursing home residents that investigated risk factors for prevalent HF in multivariable analysis. Thus, the specific pattern of risk factors in residents of nursing homes needs to be confirmed in future trial, in particular because they differ somewhat to what is described in other cohorts.

Furthermore, the diagnosis HF was not known in more than half of those having HF and a previous diagnosis of HF was not confirmed by the expert team in one third of all cases. These findings indicate an important problem as a significant number of residents obviously might not be treated correctly. This could be of significant impact to residents of nursing homes as it can be expected that symptoms are not adequately recognised and treated in a substantial number of patients. Although not prospectively investigated by randomised treatment trials in this population, this may have important impact on well-being and quality of life. Moreover, it implies a challenge to improve the diagnostic process of HF in nursing homes residents. The implementation of the structured procedure used by the expert team may improve the diagnostic process significantly. This includes echocardiography using mobile devices in residents suspected to suffer from HF. The clinical features identified in this study to be accompanied with increased risk of prevalent HF may help to detect such patients that should undergo such structured work-up.

The strength of the present study is the thorough method of data collection using an on-site integral examination of each resident. On the other hand, the participation rate was not as high as anticipated (27 %). A consideration here is that legal representatives often do not want to decide for participation on behalf of the residents, which explains the lower participation rate of psychogeriatric patients. Our findings are in line with a study conducted by Barnes [30] on HF in the elderly, where only 30 % of patients agreed to participate. Still, residents included did not differ in a clinical meaningful way from those that did not agree to participate. Therefore, it is likely that the results of this study are representative for our nursing home population in the South of the Netherlands. A 10 % missing echo values can be seen as a limitation in this study. This may result in misdiagnosis in some patients and importantly, lack of information on potential underlying causes of heart failure and left-ventricular ejection fraction. This may negatively effect quality of treatment in these patients. However, in routine clinical care, echocardiography cannot be performed in all subjects due to exactly the same reasons also found in our cohort.

Moreover, we did not investigate to what extent treatment would have changed based on more accurate diagnosis and the impact on the patients’ well-being is unknown. This is particularly true since the large treatment trials that are the basis for the treatment recommendations did not include the population of our study. Still, guideline recommendations are independent of patient’s age. Therefore, the diagnostic approach we used would at least result in treatment which is better following current guidelines. Finally, we included residents in one region only and results might be different in other countries and may depend on the structure of health care in each country. Therefore, it is important to conduct such studies in different countries as results in this regard are still very limited.

## Conclusion

HF is highly prevalent in nursing home residents with many unknown or falsely diagnosed with HF. Equal number of HF patients had reduced and preserved left-ventricular ejection fraction.
